# Dynamics alteration of the gut microbiota and faecal metabolomes in very low or extremely low birth weight infants: a Chinese single-center, prospective cohort study

**DOI:** 10.3389/fmicb.2024.1438213

**Published:** 2024-08-23

**Authors:** Ling Liu, Chaohong Chen, YeShan Li, Dang Ao, Jiayuan Wu, Nali Cai, Wen Li, Min Xiang

**Affiliations:** ^1^Department of Pediatrics, Affiliated Hospital of Guangdong Medical University, Zhanjiang, China; ^2^Clinical Research Service Center, Affiliated Hospital of Guangdong Medical University, Zhanjiang, China; ^3^Department of Orthopedics, Affiliated Hospital of Guangdong Medical University, Zhanjiang, China

**Keywords:** gut microbiota, VLBW/ELBW infants, perterm infants, steroid hormone biosynthesis, 16S rRNA sequencing

## Abstract

**Objective:**

The aim of this study is to comprehensively investigate the temporal dynamics of faecal gut microbiota and metabonomics in early postnatal with a focus on very low or extremely low birth weight (VLBW/ELBW) infants.

**Methods:**

We collected faecal samples from 157 VLBW/ELBW infants at three time points: days 1, 14, and 28 in a prospective cohort study. The faecal microbial diversity, abundance, composition, and metabolomic analyses were determined using 16S rRNA sequencing and liquid chromatography tandem mass spectrometry (LC-MS/MS). Microbiome functional analyses were conducted utilizing PICRUSt2. The ecological association networks were employed to investigate the interactions between gut microbiota and identify the core genus within 28 days of birth, as well as to unveil correlations between taxa and metabolites.

**Result:**

(1) The alpha diversity of gut microbiota significantly decreased from D1 to D28, accompanied by an interrupted trajectory lacking obligate anaerobes. At the phylum level, the 16S RNA sequencing results showed an increase in Proteobacteria and a decrease in Firmicutes and Bacteroidota from D1 to D28. At the genus level, there was a decrease in the relative abundance of *Staphylococcus*, *Acinetobacter* and *Ureaplasma*, with *Klebsiella* and *Enterococcus* emerging as the most abundant genera. (2) The analysis revealed a total of 561 metabolic markers that exhibited significant and distinct alterations between D1 and D14. (3) Ecological association networks revealed that the gut microbiota in D1 exhibited a significantly higher degree of microbial interactions compared to those in D14 and D28. Additionally, *Enterococcus*, *Klebsiella*, and *Enterobacter* were major contributors to the co-occurring network at these three time points. (4) Steroid hormone biosynthesis, including tetrahydrocortisone, androsterone glucuronide, androstenedione and etiocholanolone glucuronide, decreased within 28 days after birth.

**Conclusion:**

We have successfully demonstrated a significant dysbiosis in the gut microbiota and a subsequent decrease in its diversity within 4 weeks postpartum in VLBW/ELBW infants. Monitoring the gut microbiota of VLBW/ELBW infants and promptly rectifying dysbiosis in the early stages may represent a potential therapeutic strategy.

## Introduction

The gut microbiome, referred to as the “second genome” of humans, play a pivotal role in maintaining immune system function and overall health (Wan et al., 2020). Numerous studies have indicated that the gut microbiota actively participated in various physiological processes such as regulating host energy balance, supporting immune responses, managing energy metabolism, controlling inflammation, and promoting epithelial development (Han et al., 2022; Zhu et al., 2023). The stability and resilience of gut microbiota, however, can be influenced by various factors including dietary choices, living conditions, medication usage, illnesses, particularly the administration of antibiotics (Ho et al., 2020).

Numerous research studies consistently demonstrate a robust correlation between gut microbiota and diseases commonly observed in premature infants. Several investigations underscore the pivotal role of intestinal bacteria in the development of conditions such as necrotizing enterocolitis (NEC), early onset neonatal sepsis (EONS), feeding intolerance, and bronchopulmonary dysplasia (Collins et al., 2017; Hong et al., 2023; Jia et al., 2022). Our previous study findings indicated a significant imbalance in the composition of gut microbiota among premature infants with white matter injury. Specifically, certain groups of microorganisms such as Bacteroidetes, *Staphylococcus*, and *Acinetobacter* potentially contributes to impairing the brain’s white matter through downregulation of the bile acid biosynthesis pathway (Liu et al., 2023). A considerable proportion of premature infants in the neonatal intensive care unit (NICU) are administered antibiotics shortly after birth, potentially leading to alterations in the early-life development of their gut microbiome and impacting various metabolic pathways (Bender et al., 2021; Russell et al., 2021). The gut microbial composition of preterm babies may exhibit increased vulnerability to disruption caused by external factors such as postnatal antibiotic administration, mode of delivery, and feeding (Wang et al., 2020).

While previous longitudinal studies had documented the dynamic changes in gut microbiota among preterm infants of varying gestational age, conflicting findings had emerged (Hong et al., 2023; Jia et al., 2022). Our study also identified distinct characteristics in the faecal microbiome and metabolome of preterm infants compared to term infants, particularly those born before 32 weeks. The early postnatal period for VLBW/ELBW infants represents a unique biological process wherein disruptions in microbial colonization can have long-lasting and potentially irreversible effects on ecological succession events. However, there has been limited research conducted on the dynamic changes of gut microbiome and critical metabolites derived from gut microbiota in VLBW/ELBW infants during the early postnatal period. In this study, we aimed to investigate the sequential alterations in gut microbiota and associated metabolites of VLBW/ELBW infants within 28 days after birth. Additionally, we analyzed changes in the diversity of gut microbiota and investigate the crosstalk between gut microbiota and faecal metabolomic dynamics. Monitoring the gut microbiota of VLBW/ELBW infants and promptly rectifying dysbiosis in the early stages may represent a potential therapeutic strategy.

## Methods

### Participants and sample collection

The present observational cohort study was conducted at Affiliated Hospital of Guangdong Medical University between January 2022 and December 2023. Prior to sample collection, written informed consent was obtained from the guardians of each infant. The study included infants who met the following criteria: (1) gestational age at birth <32 weeks; (2) birth weight <1.5 kg; (3) admission within 24 h after birth. Participants were excluded if they met any of the following criteria: (1) death; (2) patients with congenital chromosomal abnormalities or genetic metabolic diseases; (3) use of microecological preparations such as probiotics, prebiotics and biostime.

The meconium was collected within 24 h after birth, and faecal samples were also collected on days 14 and 28 postpartum, named as D1, D14 and D28, respectively. The specific details can be found in Table 1. Faecal samples were obtained from the diapers of three different location using sterile tubes, with each tube containing approximately 0.2 g (similar in size to a soybean). All collected samples were promptly sealed, labeled, and subsequently stored at −80°C in the laboratory for genetic analysis.

**Table 1:** Baseline characteristics of participants included in the study, *N* = 157.

Characteristics	No. (%) or mean (SD)
<28 week (*n*, %)	15 (9.5%)
28^+1^–30^+6^ week (*n*, %)	42 (26.7%)
30^+1^–32^+6^ week (*n*, %)	100 (63.6%)
Gestational age ( $\bar{x}$ ± s, week)	30.4 ± 0.18
Birth weight ( $\bar{x}$ ± s, kg)	1.27 ± 0.018
Male (*n*, %)	87 (55.4%)
Caesarean section (*n*, %)	95 (60.5%)
SGA (*n*, %)	35 (22.2%)
GDM (*n*, %)	32 (20.3%)
Prenatal antibiotic use (*n*, %)	35 (22.2%)
Preeclampsia (*n*, %)	39 (24.8%)
Premature rupture of fetal membranes (*n*, %)	20 (12.7%)
Prenatal hormones (*n*, %)	87 (55.4%)
Duration of antibiotic use after birth (IQR, day)	6 (0, 46)
Positive pressure ventilation time (IQR, day)	8 (0, 60)
Sepsis (*n*, %)	58 (36.9%)
Mixed feeding (*n*, %)	137 (87.2%)
Total intestinal feeding time (IQR, day)	14 (5, 53)
BPD (*n*, %)	32 (20.3%)

SGA, small for gestational age; BPD, bronchopulmonary dysplasia; GDM, gestational diabetes mellitus.

### DNA extraction, library preparation and sequencing

The E.Z.N.A.^®^ soil DNA Kit (Omega Bio-tek, Norcross, GA, United States) was utilized to extract genomic DNA from the samples following the manufacturer’s instructions. The ABI GeneAmp^®^ 9700 PCR thermocycler (ABI, CA, United States) was employed for amplifying the hypervariable region V3–V4 of the bacterial 16S rRNA gene using primer pairs 338F (5′-ACTCCTACGGGAGGCAGCAG-3′) and 806R (5′-GGACTACHVGGGTWTCTAAT-3′). The amplification process involved an initial denaturation step at 95°C for 3 min, followed by a series of cycles consisting of denaturing at 95°C for 30 s, annealing at 55°C for 30 s and extension at 72°Cfor 45 s. A final single extension step was performed at 72°C for a duration of 10 min before concluding the process with cooling down to 4°C.

The amplicons were purified and combined in equal amounts before undergoing paired-end sequencing on an Illumina MiSeq PE300 platform (Illumina, San Diego, United States) following standard protocols provided by Majorbio Bio-Pharm Technology Co., Ltd. (Shanghai, China). The resulting raw reads have been deposited into the NCBI Sequence Read Archive (SRA) database under the BioProject ID PRJNA1101213.

### Species annotation

The taxonomic classification of each Operational Taxonomic Unit (OTU) representative sequence was assessed using RDP Classifier version 2.2, against the 16S rRNA gene database (e.g., Silva v138), with a confidence threshold set at 0.7. The analysis of gut microbiota’s bioinformatics was conducted on the Majorbio Cloud platform.^1^ Utilizing information from OTUs, we calculated rarefaction curves and alpha diversity indices such as observed OTUs, Chao1 richness, and Shannon index using Mothur v1.30.1 (Schloss et al., 2009). To examine beta diversity, principal coordinate analysis (PCoA) based on Bray–Curtis dissimilarity measure was performed employing the Vegan v2.5-3 package. The Linear discriminant analysis Effect Size (LEfSE) method was employed with an LDA score threshold of 4.0 to identify significant biomarkers across different taxonomic levels, ranging from phylum to genus level. Additionally, we assessed the gut microbiome health index (GMHI) and gut microbiota health disorder index (MDI). The network diagram was constructed based on Spearman correlation analysis, considering species interactions in the sample with a correlation coefficient value ≥0.5 and *p* < 0.05. Moreover, function prediction was performed using PICRUSt2.0 software (Phylogenetic Investigation of Communities by Reconstruction of Unobserved States). Furthermore, the microbial ecological network was analyzed using Networkx (version v1.11) through Spearman correlation analysis.

### Metabolite extraction and LC-MS/MS analysis

A 50 mg sample was introduced into a centrifuge tube with a capacity of 2 mL, followed by the addition of a grinding bead measuring 6 mm in diameter. For metabolite extraction, an extraction solution consisting of methanol and water in a ratio of 4:1 (v:v) was used, containing an internal standard (L-2-chlorophenylalanine) at a concentration of 0.02 mg/mL. The samples were subjected to freezing at −20°C for a duration of 30 min, followed by centrifugation at 13,000 g and 4°C for 15 min. Subsequently, the supernatant was transferred to an injection vial for LC-MS/MS analysis.

The LC-MS/MS analysis of the sample was conducted using a Thermo UHPLC-Q Exactive HF-X system equipped with an ACQUITY HSS T3 column (100 mm × 2.1 mm i.d., 1.8 μm, Waters, United States) at Majorbio Bio-Pharm Technology Co., Ltd. in Shanghai, China. The Progenesis QI software was utilized for pretreating the LC/MS raw data, resulting in a three-dimensional data matrix exported in CSV format. Additionally, metabolites were identified through database searches primarily utilizing HMDB,^2^ Metlin,^3^ and Majorbio Database resources. Subsequently, the R package “ropls” (Version 1.6.2) was employed to conduct principal component analysis (PCA) and orthogonal least partial squares discriminant analysis (OPLS-DA). Furthermore, a 7-cycle interactive validation approach was implemented to evaluate the model’s stability.

The significantly different metabolites were identified based on their Variable Importance in the Projection (VIP) values >1 and *p* < 0.05. Metabolic enrichment analysis, followed by Benjamini–Hochberg correction and pathway analysis using the KEGG database,^4^ was performed to classify these differential metabolites into their respective biochemical pathways. Enrichment analysis using the Python package “scipy.stats”^5^ was conducted to identify the most relevant biological pathways associated with the experimental treatments. Spearman correlation coefficient (≥0.6, *p* < 0.5) was utilized for network analysis to establish connections between metabolites and species variables.

### Bioinformatics and statistical analysis

The data analysis was performed using IBM SPSS Statistics 26 and R software (Version 2.15.3). Categorical variables were expressed as frequencies and percentages [*n* (%)], while continuous variables, whether normally or non-normally distributed, were presented as means ± standard deviation or medians with interquartile ranges (IQR). The normality of distribution comparisons between groups was assessed using the Student’s *t*-test. For groups with non-normal distributions, such as alpha diversity, relative abundance of bacteria, GMHI, and MDI, either the Mann–Whitney *U* test or the Wilcoxon rank sum test was employed for analysis. The differences in categorical data were assessed using statistical tests such as chi-squared or Fisher’s exact test. Furthermore, the association between gut microbiota at the genus level and metabolite parameters was evaluated through the Spearman correlation coefficient test. Statistical significance was determined based on a two-sided *p*-value threshold of less than 0.05. ^*^p < 0.05, ^**^p < 0.01, and ^***^p < 0.001.

## Results

### Study cohort description

During the period from January 2022 to December 2023, a total of 173 eligible subjects were enrolled at the Affiliated Hospital of Guangdong Medical University. After excluding cases involving 16 deaths or discontinuation of treatment by their families, the study ultimately included 157 individuals. The baseline characteristics of these patients are summarized in Table 1. Among them, there were 87 (55.4%) male participants with an average gestational age of 30.4 ± 0.18 weeks and an average birth weight of 1.27 ± 0.018 kg upon admission.

### Alterations in the gut microbiota’s diversity and composition over a period of time

**Figure 1:**
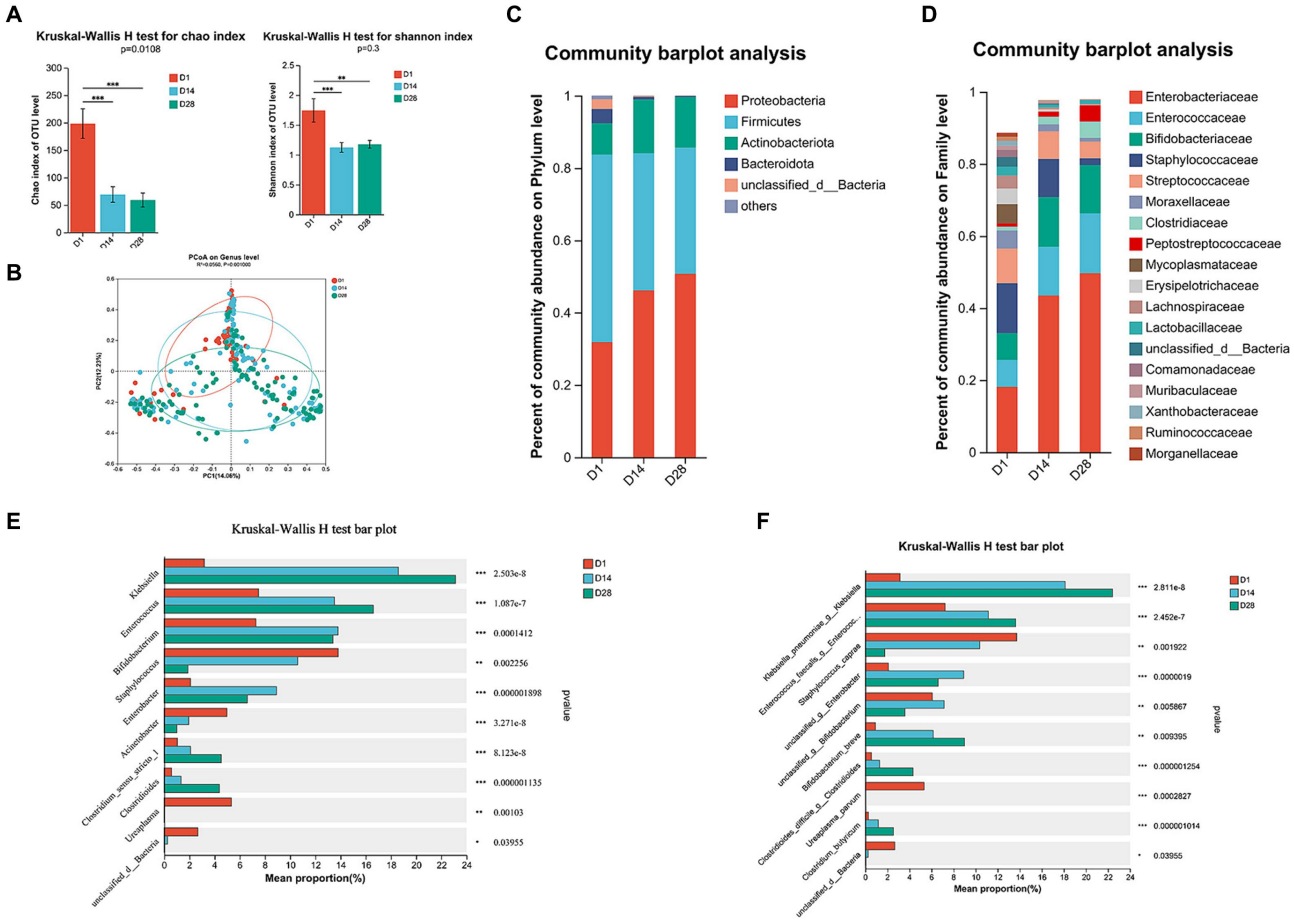
Temporal dynamics of gut microbiota composition through 16S rRNA gene sequencing. **(A)** The alpha diversity of gut microbiota was assessed using the Chao1 and Shannon indices. **(B)** The beta diversity of the gut microbiota was visualized by principal coordinates analysis (PCoA) plot based on Bray–Curtis analysis. **(C)** Relative abundance of microbial composition at the phylum level. **(D)** Family-level relative abundance of microbial composition. **(E)** Significant differences were observed in the genus-level composition of gut microbiota. **(F)** Abundance profiles at the species level were determined for all time points. Differences between groups were assessed using the Wilcoxon rank sum test. The *p*-value was corrected for multiple comparisons using the Benjamini–Hochberg method. ^*^p < 0.05, ^**^p < 0.01, and ^***^p < 0.001.

The alpha diversity indices, including Chao, Shannon, and ACE indices, exhibited a significant distinction (Wilcoxon test, *p* < 0.05; Figure 1A; Supplementary Figure S1A), indicating that the abundance and diversity of gut microbiota were notably higher in D1 compared to D14 and D28. The alpha diversity metrics did not show any significant variation at D14 and D28. The PCoA analysis based on Bray–Curtis distances revealed distinct segregation at the genus level over time, specifically at sampling timepoints D1, D14, and D28 (*R*^2^ = 0.056, *p* = 0.001; Figure 1B). The ANOSIM/Adonis test demonstrated a significant and substantial divergence in the overall community structure between time points D1 versus both D14 and D28 (*p* < 0.001; Supplementary Figures S1B–D), indicating that microbial composition at D1 differs from days 14 and 28. The diversity of VLBW/ELBW exhibited significant fluctuations, gradually decreasing postnatally.

The temporal dynamics of gut microbiota composition were investigated across various taxonomic levels, ranging from phylum to genus. VLBW/ELBW infants exhibited a conspicuous absence of obligate anaerobes within the first 28 days after birth. Compared to D14 and D28, the relative abundance of Firmicutes, Bacteroidota, Fusobacteriota, and Patescibacteria at the phylum level was significantly increased in D1 (Figure 1C; Supplementary Figure S2A). Furthermore, there was an increase in the prevalence of Proteobacteria, the most dominant phylum, from D1 to D28. Conversely, Firmicutes, Bacteroidota and Fusobacteriota exhibited a decrease in relative abundances from D1 to D28 (Figure 1C; Supplementary Figures S2B,C). At the family level, temporal changes in the relative increased abundance dominant bacteria of Enterobacteriaceae and Enterococcaceae (≥30% relative abundance) were observed from D1 to D28. Furthermore, there was a tendency towards decreased proportions of Staphylococcaceae and Moraxellaceae over time (Figure 1D; Supplementary Figure S2D). Conversely, Bifidobacteriaceae exhibited a higher abundance in D14 and D28 compared to D1 (Figure 1D; Supplementary Figure S2D).

**Figure 2:**
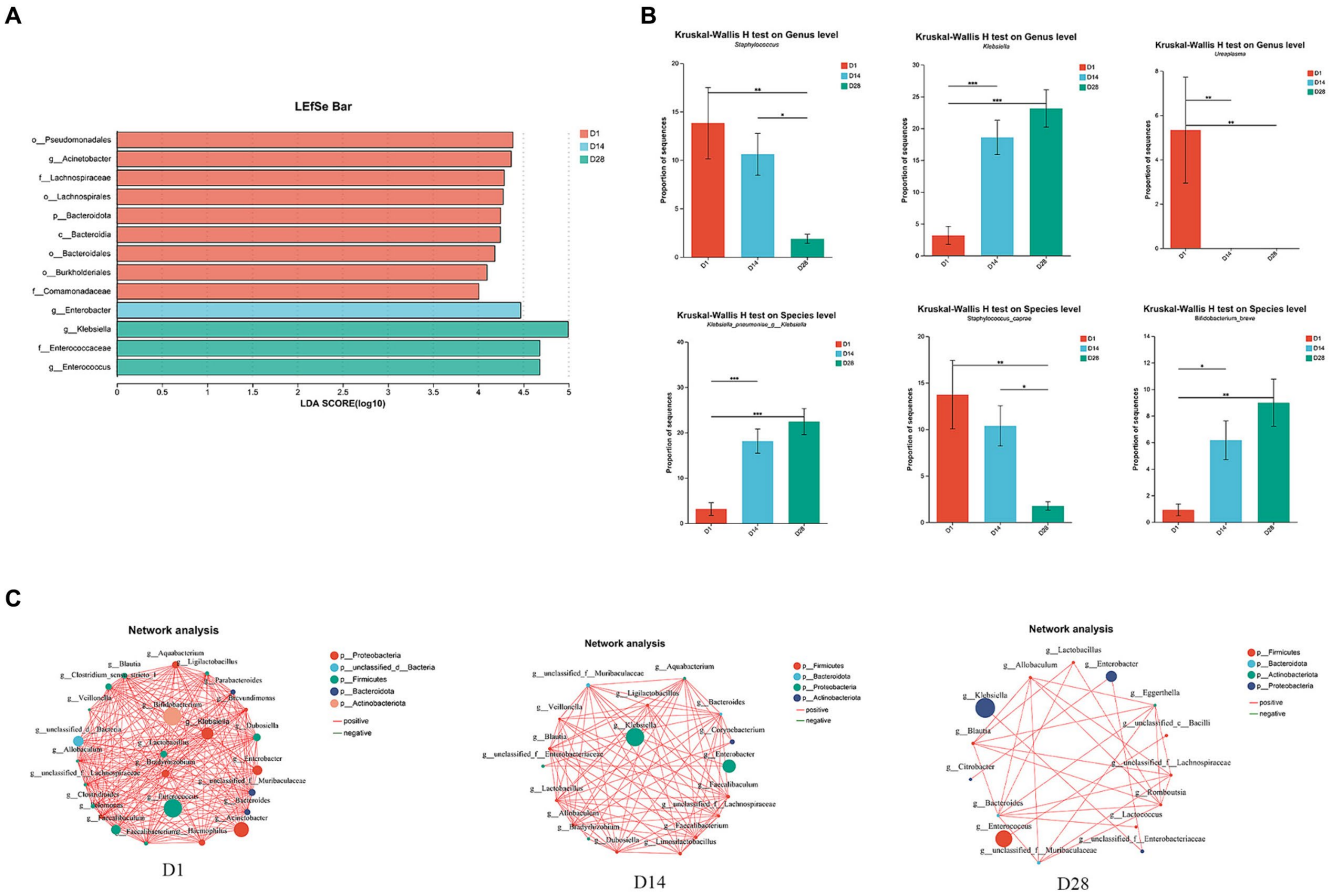
Overall variations in the gut microbiota across time points D1, D14, and D28. **(A)** LEfSe analysis was conducted to identify the highly significant microbiota, ranging from phylum to genus level, using a threshold of LDA (LDA ≥4). **(B)** The relative abundance of *g__Staphylococcus*, *g__Klebsiella*, *Ureaplasma*, Klebsiella_pneumoniae_g__Klebsiella, Staphylococcus_caprae, and *Bifidobacterium breve* varied significantly. **(C)** Ecological association networks were constructed to analyze the gut microbiota at the genus level on days 1, 14, and 28. Only *p*-values <0.05 and |*r*| *≥* 0.5 are displayed in the network. Each node represented a genus. Red line: positive correlation; green line: negative correlation. The number of connecting lines increases as the relationship between genera becomes closer. Each node is assigned a color according to its corresponding phylum.

At the genus level, D1 exhibited a prevalence of *Staphylococcus*, *Acinetobacter*, *Ureaplasma* and *Faecalibaculum*. In contrast, D14 was characterized by the presence of *Enterobacter* genera. Additionally, D28 showed a high abundance of *Clostridium_sensu_stricto_1* and *Clostridioides* (Figures 1E, 2B). During the period from D1 to D28, we noted a significant augmentation in the abundance of specific gut microbiota including *Klebsiella*, *Enterococcus*, *Clostridium_sensu_stricto_1* and *Clostridioides*. In contrast, there was a notable decline in levels of *Staphylococcus*, *Acinetobacter* and *Ureaplasma*. The LEfSe analysis (Figure 2A) revealed that in D1, the most predominant bacterial taxa were o_Pseudomonadales, *g_Acinetobacter*, f_Lachnospiraceae, o_Lachnospirales and p_Bacteroidota (Figure 2A, LDA ≥4). Importantly, a higher abundance of *g_Enterobacter* was observed in D14, while increased abundances of *g_Klebsiella*, f_Enterococcaceae and *g_Enterococcus* were found in D28.

### Analysis of ecological association networks in gut microbiota

Ecological association networks primarily reflect species correlations across various taxonomic levels. Therefore, we conducted Spearman’s correlation analysis and constructed ecological association networks for the top 30 most abundant genera to elucidate the interactions between gut microbiota and identify the core genera within 28 days of birth (Figure 2C). The results revealed significant variations in the gut microbiota, with D1 exhibiting the highest level of microbial interactions compared to D14 and D28. Within the microbial community, *Enterococcus*, *Bifidobacterium*, *Acinetobacter* and *Klebsiella* emerged as dominant species in D1. Interestingly, both *Klebsiella* and *Enterobacter* demonstrated a substantial advantage in D14 and D28. During the early postnatal period, time-dependent changes were observed in the ecological association networks among gut microbiota. The co-occurrence network was primarily influenced by *Enterococcus*, *Klebsiella* and *Enterobacter* at three distinct timepoints (Figure 2C).

### Temporal dynamics of faecal metabolomic profiles

**Figure 3:**
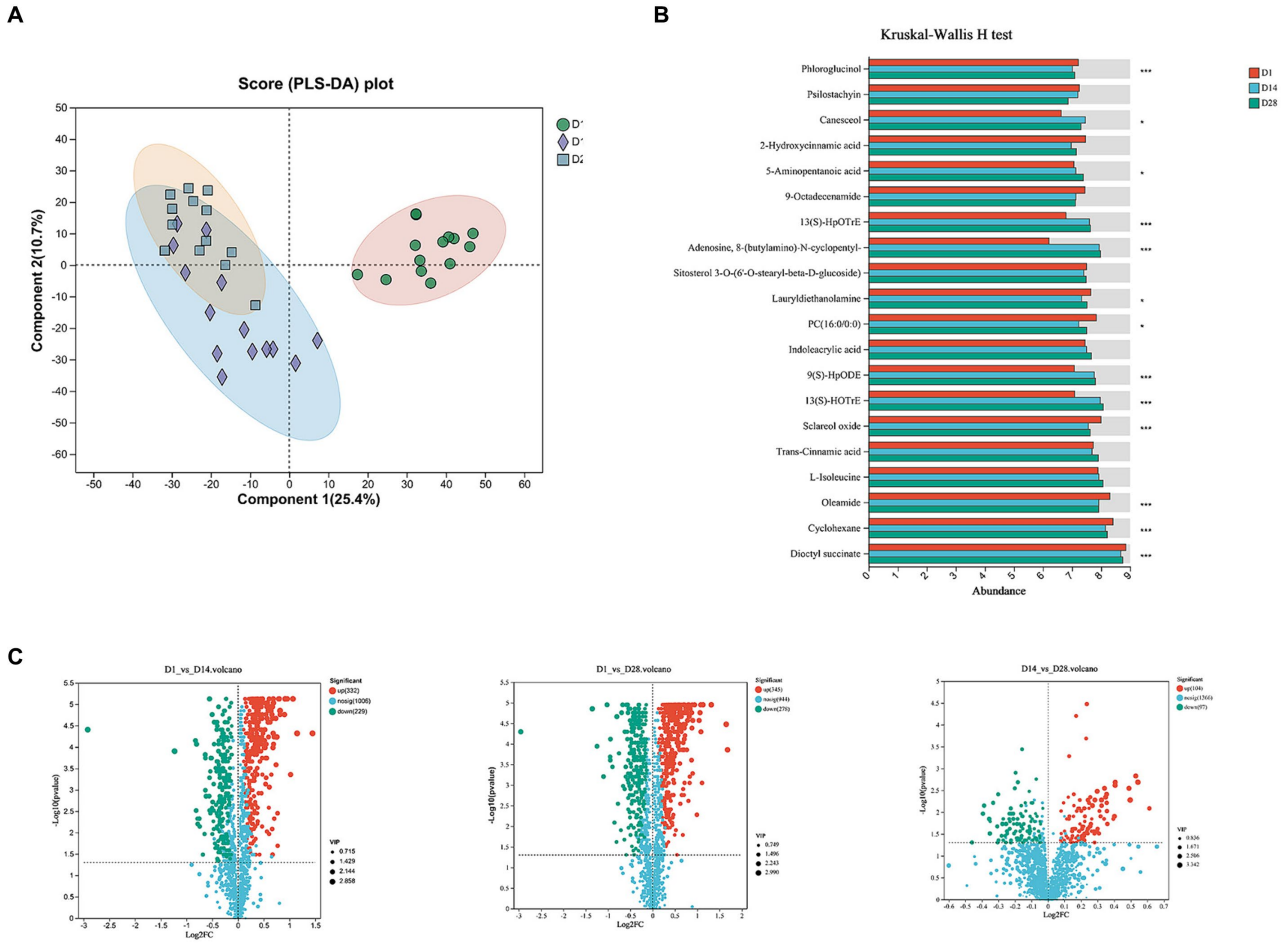
Temporal changes in faecal metabolites. **(A)** OPLS-DA score plot. **(B)** The histogram displayed the top 20 most abundant metabolites of significant importance. **(C)** The volcano plot illustrated different metabolites in positive ion compound: green indicates the down-regulated differential metabolite, while red represents the up-regulated differential metabolite.

To elucidate the metabolomic profile within 1 month postpartum, untargeted metabolomics analysis was performed on faecal samples in conjunction with 16S rRNA profiling. A total of 1,570 identifiable metabolites of positive iron and 1,086 identifiable metabolites of negative iron were determined based on a combination of statistically significant parameters from univariate analysis (*p* < 0.05) and multivariate analysis (VIP >1) with advancing gestation. The OPLS-DA analysis revealed a significant perturbation in the dynamic distributions of metabolomic data (*p* < 0.001), resulting in discernible variations in the overall faecal metabolic profiles (Figures 3A,B). A total of 561 faecal metabolites exhibited significant changes between D1 and D14, while 623 showed considerable alterations between D1 and D28. Furthermore, there were 201 metabolites that displayed notable differences between D14 and D28 (Figure 3C).

### Altered metabolic pathways and their biological significance

**Figure 4:**
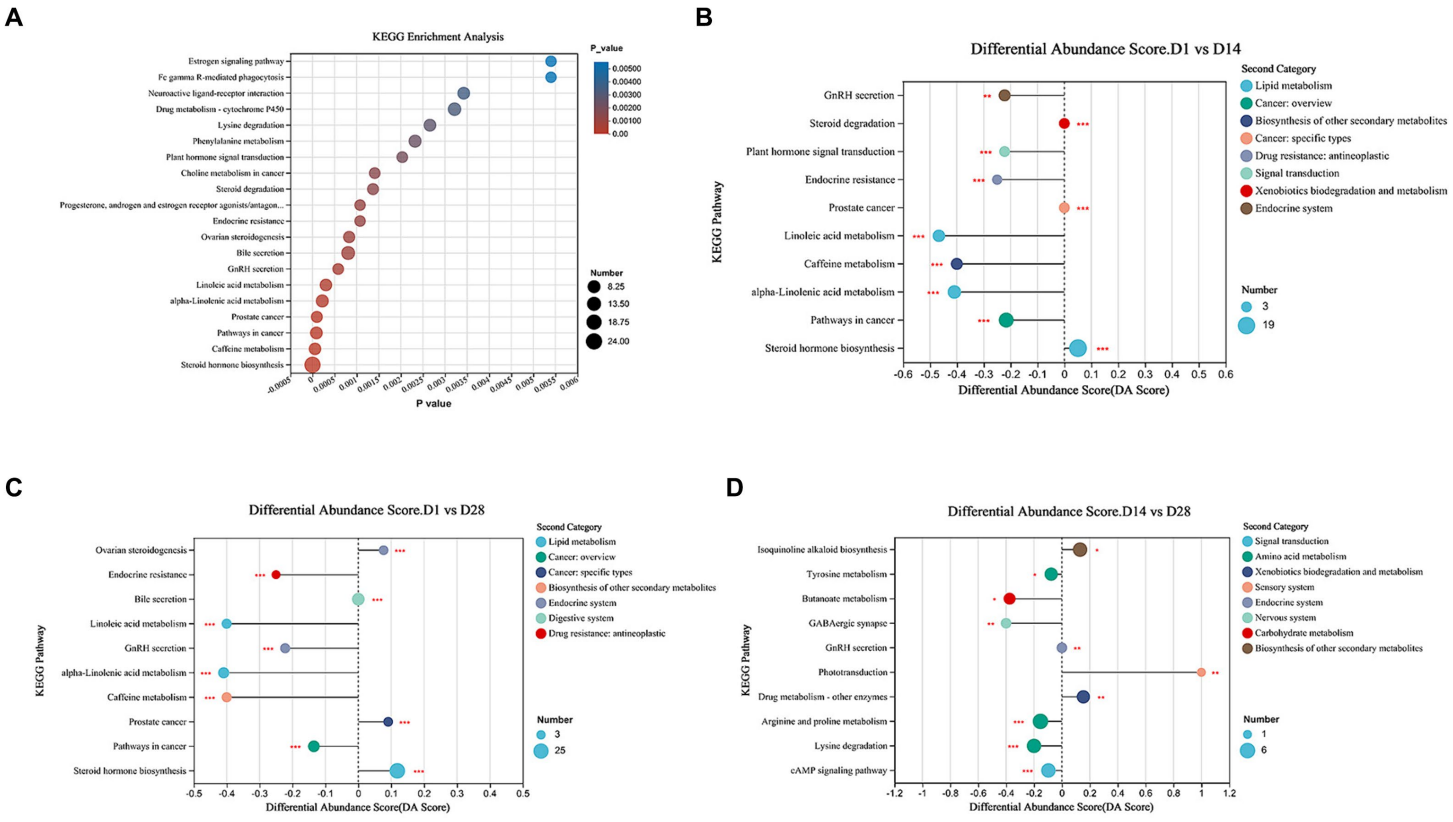
Metabolic pathways enrichment analysis. **(A)** Overview of metabolites that exhibited enrichment based on the KEGG database during early postnatal period. **(B)** Comparison of the top 10 significantly different metabolic pathways between D1 and D14. **(C)** Comparison of the top 10 significantly different metabolic pathways between D1 and D28 was conducted. **(D)** Comparison of top 10 significantly differential metabolic pathways between D14 and D28. The differential abundance (DA) score is represented on the *X*-axis, and the KEGG metabolic pathways are depicted on the *Y*-axis. A score of 1 signifies an up-regulated expression trend, whereas −1 indicates a down-regulated expression trend.

To investigate the alterations in metabolic pathways associated with differentially expressed metabolites and their biological functions, we performed an enrichment analysis using KEGG. The most prominent metabolic pathways identified included steroid hormone biosynthesis, caffeine metabolism, Pathways in cancer, prostate cancer, alpha-Linolenic acid metabolism, Linoleic acid metabolism, and GnRH secretion pathway (Figure 4A). KEGG enrichment analysis revealed significant alterations in 28 metabolic pathways between D1 and D14, with adjusted *p*-value <0.05. Compared to D14, downregulation of the GnRH signaling pathway, plant hormone signal transduction, endocrine resistance, linoleic acid metabolism, caffeine metabolism, and alpha-linolenic acid metabolism was observed in D1 (Figure 4B). Conversely, an upregulation of steroid hormone biosynthesis was evident in D1. The comparison between D1 and D28 revealed 45 perturbed metabolic pathways with significantly lower *p*-values and higher pathway impact (Figure 4C). These included down-regulated endocrine resistance, linoleic acid metabolism, alpha-linolenic acid metabolism, and caffeine metabolism; as well as up-regulated ovarian steroidogenesis and steroid hormone biosynthesis in D1 compared to D28. The metabolic pathways, including down-regulated tyrosine metabolism, butanoate metabolism, GABAergic synapse and arginine and proline metabolism, showed lower *p*-values and higher pathway impact in D14 compared to D28. Conversely, phototransduction and drug metabolism - other enzymes were up-regulated in D14 compared to D28 (Figure 4D). Overall, the metabolomics analysis revealed significant and programmed changes in both microbial community functions as gestation progressed.

### The gut microbiota correlates with faecal metabolites and inferred metabolic pathways

**Figure 5:**
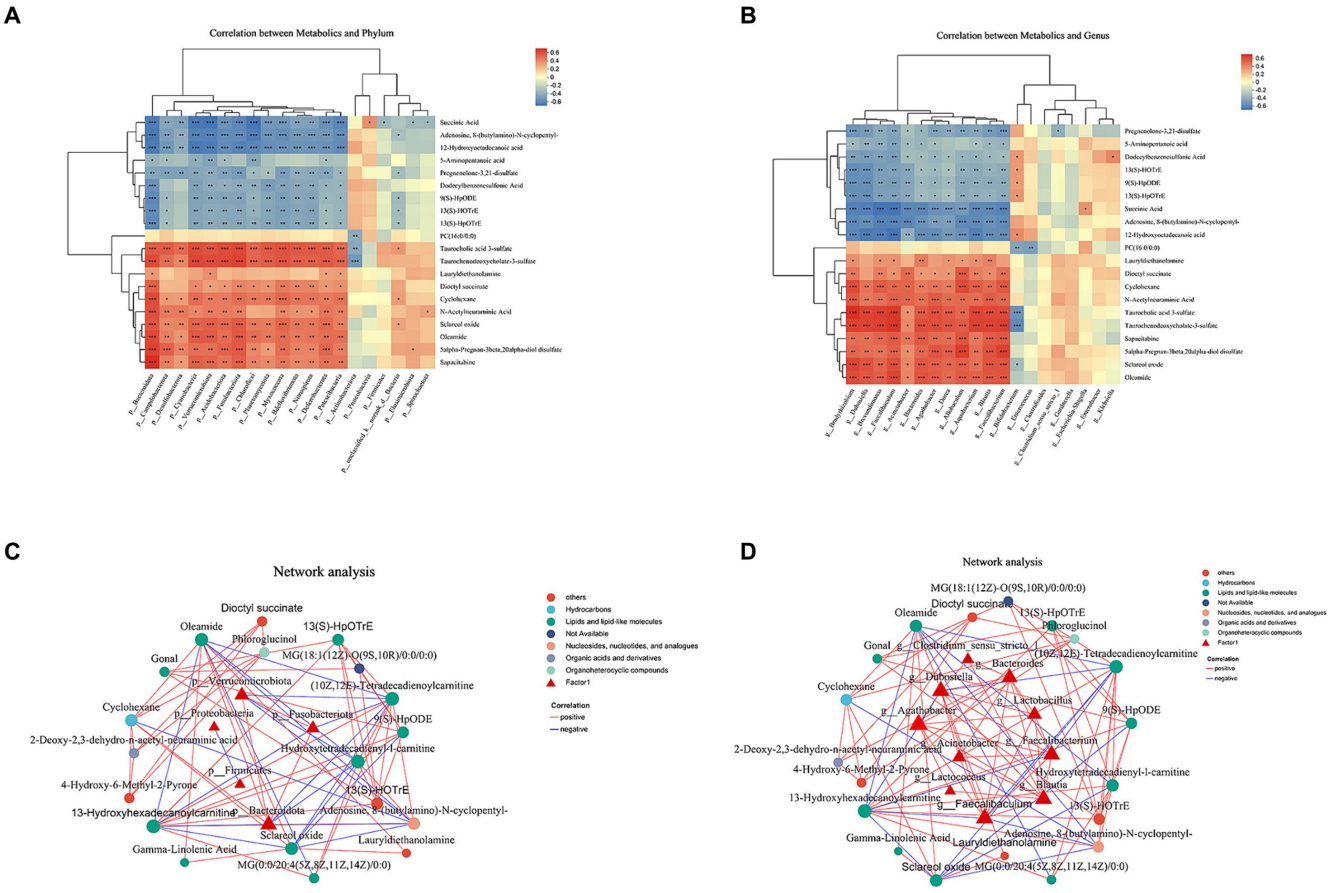
Interactions between the abundance of gut microbiota and faecal metabolites. **(A)** Heatmap illustrating the correlation coefficients between the top 20 identified metabolites in stool and the top 10 differentially abundant phyla. **(B)** Heatmap illustrating the repeated measures correlation coefficients between the top 20 identified metabolites in stool and the top 10 differentially abundant genera. Relevant correlations are denoted by a “+” symbol, accompanied by FDR-corrected *p* < 0.05. **(C)** Correlation network analysis of gut microbiota and faecal metabolites at the phylum level. **(D)** Correlation network analysis of gut microbiota and faecal metabolites at genus level.

We conducted Spearman’s correlation analysis to investigate the potential association between gut bacteria and relative concentrations of faecal metabolites (Figure 5). The phylum-level analysis revealed that p_Bacteroidota and p_Verrucomicrobiota were the primary drivers of alterations in faecal metabolite concentrations. The abundances of p_Bacteroidota exhibited inverse correlations with the concentrations of faecal succinic acid, adenosine, 8-(butylamino)-N-cyclopentyl-, 12-hydroxyoctadecanoic acid and dodecylbenzenesulfonic acid (Figures 5A,C). The abundances of p_Bacteroidota exhibited positive correlations with the concentrations of faecal Taurochenodeoxycholate-3-sulfate, cyclohexane, oleamide and sapacitabine (Figures 5A,C). The abundances of p_Verrucomicrobiota exhibited inverse correlations with the concentrations of faecal adenosine, 8-(butylamino)-N-cyclopentyl-, (10Z,12E)-tetradecadienoylcarnitine and hydroxytetradecadienyl-l-carnitine (Figures 5A,C). Significantly, the genera *Faecalibaculum* and *Dubosiella* emerged as the primary drivers of alterations in faecal metabolite concentrations. Notably, variations in the microbiota exhibited a robust correlation with changes in lipid and lipid-like molecule levels. We investigated the associations between taxa and metabolites, revealing a negative correlation between the abundance of *Faecalibaculum*, *Faecalibacterium* and *Dubosiella* with the concentration of (10Z,12E)-tetradecadienoylcarnitine and 13-hydroxyhexadecanoylcarnitine. Additionally, we observed a negative correlation between the abundance of *Faecalibaculum* and adenosine, 8-(butylamino)-N-cyclopentyl- (Figures 5B,D).

## Discussion

This finding holded great significance as it established a fundamental understanding of the pivotal role played by the overall gut ecosystem in VLBW/ELBW infants. By integrating 16S rRNA gene sequencing with LC-MS/MS metabolomics, we observed substantial fluctuations in both the alpha diversity and overall composition and metabolic function from day 1 to day 28. Furthermore, the ecological networks effectively captured the developmental trajectory of gut microbes from day 1 to day 28. The identification of these early indicators within the gut microbiota had provided innovative perspectives on tailoring treatment approaches for VLBW/ELBW infants.

The gut microbiota of preterm infants exhibits significant dissimilarities compared to those full-term, characterized by reduced microbial diversity and increased colonization of pathogenic microorganisms (Westaway et al., 2022; Zhang et al., 2022). Our study has demonstrated that the gut microbial richness and diversity of D1 were significantly higher than those of D14 and D28, and the diversity of VLBW/ELBW gradually decreased after birth. Based on these findings, we hypothesized that most VLBW/ELBW infants may have initiated prophylactic antimicrobial treatment within 14 days. The microbial diversity did not revert to the baseline levels within 28 days postpartum. Jia et al. (2022) discovered a higher alpha diversity of meconium in the extremely preterm group compared to the very preterm, moderate to late preterm, and term groups. Furthermore, our previous research also found that both ACE and Chao indices were significantly elevated in in less than 32 weeks gestational age group compared to term infant. The neonatal gut microbiota exhibits remarkable dynamism and undergoes evolutionary changes throughout the first 3 years of life (Hui et al., 2021).

All the analyses conducted at multiple levels consistently supported the hypothesis that there were significant shifts in signatures from D1 to D28. The analysis of our data revealed an observed increase in the prevalence of Proteobacteria within the gut microbiome of VLBW/ELBW infants from D1 to D28. In contrast, there was a decline in the relative abundances of Firmicutes, Bacteroidota, and Fusobacteriota during this time period. Furthermore, the high abundance of Proteobacteria had been proposed to signify dysbiosis-related microbial characteristics and reflected the unstable structure of the intestinal microbial community (Shin et al., 2015). Previous studies had demonstrated that the gut microbiota of all infants is initially dominated by Proteobacteria, which gradually decreased from birth up to 24 months of age, while Bacteroidetes and Firmicutes exhibited a gradual increase (Yap et al., 2021). The findings of our research, however, contradicted the prevailing notion. Our study necessitates an extended follow-up period to further validate the enduring alterations in the gut microbiota. Our previous study found that increased relative abundances of γ-proteobacteria and *Escherichia-Shigella* and decreased abundance of *Bacteroides* in meconium were associated with an increased risk of feeding intolerance in VLBW/ELBW infants (Liu et al., 2022).

Our findings suggested an increase in the abundance of specific gut microbiota, including *Klebsiella*, *Enterococcus*, *Clostridium_sensu_stricto_1* and *Clostridioides*, from D1 to D28. Conversely, *Staphylococcus*, *Acinetobacter* and *Ureaplasma* demonstrated a decrease from D1 to D28. Our previous investigation had demonstrated that inhibiting the initial abnormal colonization of *Staphylococcus* and *Acinetobacter* in preterm neonates holded potential to enhance the efficacy of interventions aimed at preventing and managing white matter injury in premature infants (Liu et al., 2023). The presence of *Klebsiella* in faecal samples obtained from premature infants showed a significant increase within the first 2 weeks after birth (Yap et al., 2021). Significantly, the overgrowth of *Klebsiella* in the gastrointestinal tract exhibits a strong predictive value for brain injury (Seki et al., 2021). Our findings revealed a progressive increase in the abundance of Klebsiella_pneumoniae_g__Klebsiella, Enterococcus_faecalis_g__Enterococcus, Clostridioides_difficile_g__Clostridioides, and Bifidobacterium_breve as gestation advanced. Conversely, Staphylococcus_caprae and Ureaplasma_parvum exhibited a declining trend. The increased abundance of *K. pneumoniae* was found to be significantly associated with the risk of necrotizing enterocolitis (NEC) in preterm infants (Dobbler et al., 2017).

The ecological association networks exhibited a higher number of microbial correlations in D1 compared to D14 and D28. We further identified three types of facultative anaerobes, namely *Enterococcus*, *Klebsiella* and *Enterobacter*, as the primary contributors to the co-occurrence network at three different time points. Certain dominant genera identified in D1, such as *Bifidobacterium* and *Acinetobacter*, were absent at D14 and D28. The observed disparity in ecological networks indicates the instability of microflora in VLBW/ELBW infants. *Enterococcus* is recognized for its ability to express metalloproteases, thereby compromising the integrity of the intestinal barrier and facilitating translocation into the bloodstream in susceptible hosts, leading to inflammatory responses (Pan et al., 2024). *Enterococcus*, as a potential pathogen, facilitated infection in various sites such as the urinary tract, wounded epithelium, heart, and bloodstream due to its ability to survive in an antibiotic-rich harsh environment (Zhang R. et al., 2023). Jia et al. (2022) demonstrated variations in the duration of colonization by opportunistic pathogens such as *Klebsiella* and *Enterococcus*, with different dominant colonization patterns observed across gestational age groups. Newborn gut is initially characterized by aerobic conditions, which gradually transition to anaerobic environments. This transition facilitates the establishment of strict anaerobes such as *Bifidobacterium*, *Clostridium*, and *Bacteroides* during the process of gut colonization (Arrieta et al., 2014; Matamoros et al., 2013). However, no significant alteration was observed in VLBW/ELBW infants within the first 28 days. During the initial 28 days of life, the gut microbiota in VLBW/ELBW infants exhibited remarkable instability and potential pathogenicity.

The metabolic pathways in VLBW/ELBW infants exhibited a down-regulation of the GnRH signaling pathway, plant hormone signal transduction, endocrine resistance, linoleic acid metabolism, caffeine metabolism and alpha-Linolenic acid metabolism within 28 days of birth. Additionally, there is an up-regulation of steroid hormone biosynthesis. Steroid hormones are crucial for the regulation of hydration and sodium levels, metabolic processes, stress management, as well as the preservation of sexual differentiation and reproductive functions (Zhang Y. et al., 2023). The exposure of PM2.5 to the gastrointestinal tract induces systemic metabolic alterations by perturbing the gut microbiome and disrupting pathways involved in steroid hormone biosynthesis metabolism (Zhang Y. et al., 2023). In a predictive analysis, the potential carcinogenic pathways identified were microbial steroid hormone biosynthesis, mineral absorption, and retinol metabolism (Kalinen et al., 2024). From the perspective of host metabolism, *Faecalibaculum* and *Dubosiella* may play pivotal roles within a 28-day timeframe.

We explored correlations between taxa and metabolites, and found the abandunce of the *Faecalibaculum*, *Faecalibacterium* and *Dubosiella* was negatively correlated with the (10Z,12E)-tetradecadienoylcarnitine and 13-hydroxyhexadecanoylcarnitine concentration, and the abandunce of the *Faecalibaculum* was negatively correlated with Adenosine, 8-(butylamino)-N-cyclopentyl-. The involvement of *Faecalibaculum* has been implicated in the pathogenesis of diabetes and obesity (Yan et al., 2022). The study conducted by Li et al. (2022) demonstrated a positive correlation between the presence of *Enterococcus*, and *Faecalibaculum* species, as well as intestinal inflammation involving NF-κB and STAT3 signaling pathways.

Nevertheless, it is important to acknowledge certain constraints in our study. Firstly, the research was conducted solely at a single center, necessitating the need for validation across multiple cohorts. Secondly, the duration of sample collection in our study was limited to 28 days. Although high-throughput sequencing was utilized in this study, a more comprehensive analysis would require high-resolution shotgun metagenomic sequencing for greater accuracy and depth of information. Another limitation lied in the lack of detailed documentation regarding feeding practices (breastfeeding, formula, or mixed) and specific administration of antibiotics among preterm infants. However, larger cohorts would potentially unveil additional associations between microbes and metabolites. In the future, we aim to further extend both the follow-up duration and sample size.

## Conclusion

This study elucidated temporal dynamics in the gut microbiota and faecal metabolites during the first 28 days after birth of VLBW/ELBW infants. In addition to distinct dynamics of gut microbiota, the metabolite profiles exhibited significant alterations from D1 to D28, emphasizing the necessity for tailored microbiota-associated treatments during different postnatal periods.

## Data Availability

The datasets presented in this study can be found in online repositories. The names of the repository/repositories and accession number(s) can be found at: https://www.ncbi.nlm.nih.gov/, BioProject: PRJNA1101213.

## Footnotes

^1^ https://cloud.majorbio.com

^2^ http://www.hmdb.ca/

^3^ https://metlin.scripps.edu/

^4^ http://www.genome.jp/kegg/

^5^ https://docs.scipy.org/doc/scipy
