# Supplementary figures and images for: Dynamics alteration of the gut microbiota and faecal metabolomes in very low or extremely low birth weight infants: a Chinese single-center, prospective cohort study
Source: Front Microbiol. 2024 Aug 23;15:1438213. doi: 10.3389/fmicb.2024.1438213 (PMC11377216; doi:10.3389/fmicb.2024.1438213)

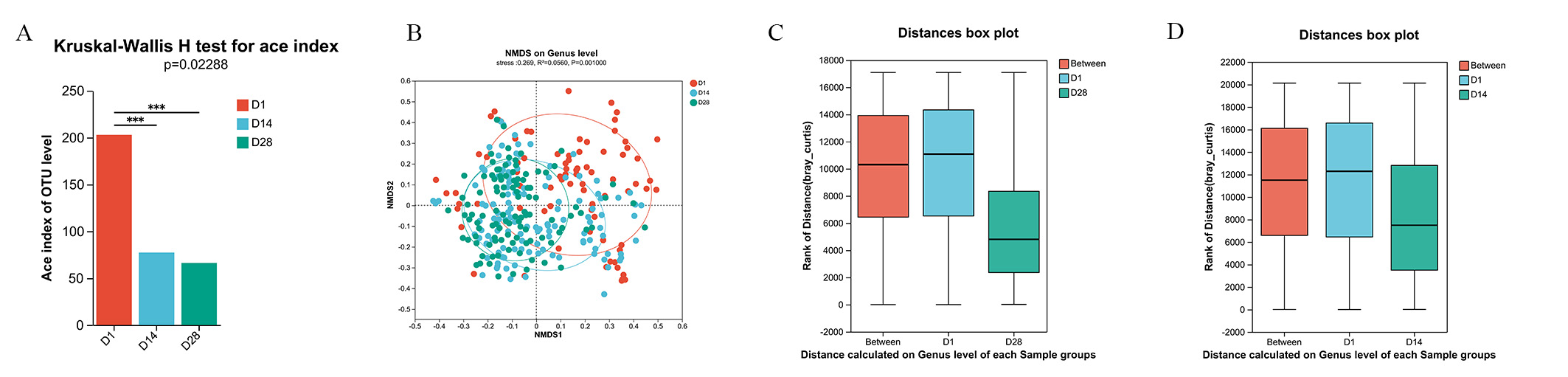

Supplement: Supplementary file 1 [file Image_1.JPEG]

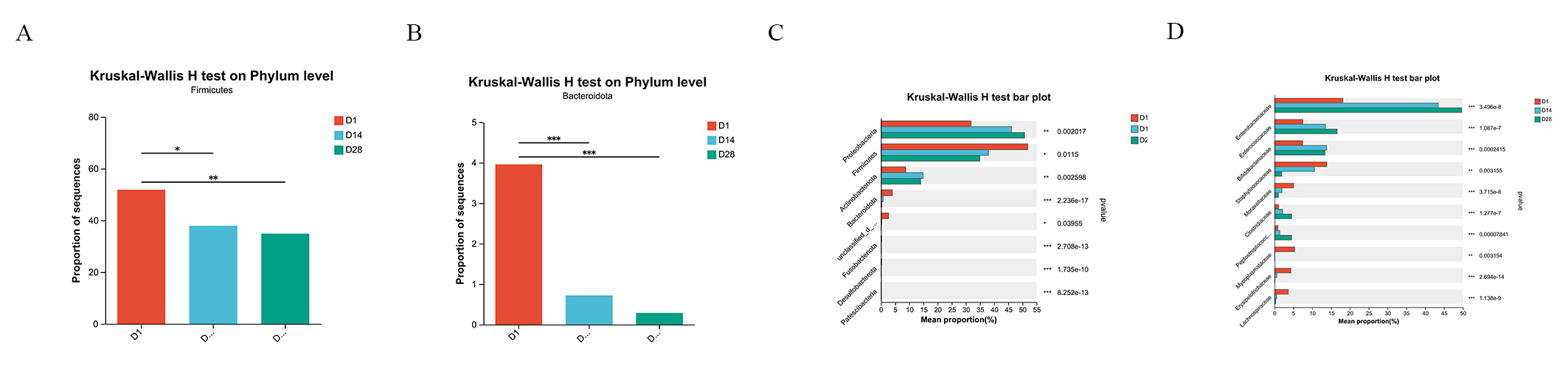

Supplement: Supplementary file 2 [file Image_2.JPEG]
